# Die Relevanz von Sozialkapitalindikatoren für die Mitgliederbindung in Zeiten von Corona

**DOI:** 10.1007/s12662-021-00793-9

**Published:** 2022-01-05

**Authors:** Ulrike Burrmann, Stephan Sielschott, Sebastian Braun

**Affiliations:** Institut für Sportwissenschaft, Humboldt-Universität zu Berlin, Unter den Linden 6, 10099 Berlin, Deutschland

**Keywords:** Bindung, Geselligkeitsorientierung, Krisenmanagement, Reziprozitätsnormen, Sportvereine, Vertrauen, Commitment, Sociability orientation, Crisis mangement, Norms of reciprocity, Sports clubs, Trust

## Abstract

Bereits vor der Corona-Pandemie berichteten immer mehr Sportvereine von zurückgehenden Mitgliederzahlen, episodischen Engagements und Schwierigkeiten bei der Gewinnung und Bindung von freiwillig Engagierten. Angesichts der komplexen Einschränkungen der Sportvereins- und geselligen Aktivitäten während der Corona-Pandemie stellt sich die Frage, wie diese außergewöhnliche soziale Situation die Mitgliederbindung in Vereinen beeinflusst hat und welche Bedeutung dem Sozialkapital zukommt. Der vorliegende Beitrag, in dem die Mitgliederbindung nach der Corona-Pandemie abgeschätzt werden soll, greift eine Forschungslücke auf, wobei v. a. die Relevanz von verschiedenen Sozialkapitalindikatoren betrachtet wird. Basis der empirischen Analysen bildet eine repräsentative Bevölkerungsbefragung, die Ende 2020/Anfang 2021 durchgeführt wurde. Die Mitgliederbindung der Sportvereinsmitglieder ist nach wie vor recht hoch. Sozialkapitalindikatoren, die sich eng auf den Sportverein beziehen, erklären wenig zur Vorhersage der Mitgliederbindung. Neben den im Verein wahrgenommenen Reziprozitätsnormen erhöhen Hilfsbereitschaft und Ingroup-Vertrauen die Bindung an den Verein, während Geselligkeitsorientierung und Outgroup-Vertrauen die Wahrscheinlichkeit verringern, auch nach der Pandemie noch Mitglied im Verein zu sein. Unter Einbeziehung aller Prädiktoren erhöht sich die Modellgüte auf knapp 26 %. Neben wenigen soziodemographischen Merkmalen tragen v. a. mitgliedschaftsbezogene Merkmale (z. B. Krisenmanagement des Vereins) zur Varianzaufklärung bei. Hilfsbereitschaft, Outgroup-Vertrauen und tendenziell auch Geselligkeitsorientierung stellen im Gesamtmodell die einzigen bedeutsamen Sozialkapitalindikatoren dar, wenngleich sie mit beachtlichen Gewichten zur Varianzaufklärung beitragen.

## Abstract (en)

Even before the corona pandemic, more and more sports clubs reported declining memberships, episodic engagement, and difficulties in recruiting and retaining volunteers. Given the complex constraints on sports clubs and social activities during the corona pandemic, the question arises as to how this exceptional social situation affected membership retention in clubs and the importance of social capital. This paper addresses a research gap by estimating membership commitment in the aftermath of the corona pandemic, focusing on the relevance of different social capital indicators. The empirical analyses are based on a representative population survey conducted in late 2020/early 2021. Membership retention of the sports club members remains quite high. Social capital indicators that are narrowly related to the sports club explain little in predicting member retention. In addition to perceived norms of reciprocity in the club, helpfulness and ingroup trust increase retention, while sociability orientation and outgroup trust decrease the likelihood of still being a member of the club after the pandemic. When all predictors are included, the model goodness of fit increases to almost 26%. In addition to a few sociodemographic characteristics, membership-related characteristics (e.g., crisis management of the association) contribute to the variance clarification. Willingness to help, outgroup trust, and a tendency towards sociability are the only significant social capital indicators in the overall model, although they contribute with considerable weights to the variance explanation.

Bereits vor der Corona-Pandemie berichteten immer mehr Sportvereine von zurückgehenden Mitgliederzahlen, episodischen Engagements und Schwierigkeiten bei der Gewinnung und Bindung von freiwillig Engagierten (Breuer, Feiler, & Rossi, 2020; Priemer, Krimmer, & Labigne, 2017; Simonson, Kelle, Kausmann, & Tesch-Römer, 2021; mit Blick auf das ehrenamtliche Engagement: Breuer, 2017; Perck, Van Hoecke, Westerbeek, & Breesch, 2016; Pitsch & Emrich, 1997; Thieme, Liebetreu, & Wallrodt, 2017; Van der Roest, van Kalmthout, & Meijs, 2016). Für die abnehmende Bindungsbereitschaft der Mitglieder werden in der Literatur unterschiedliche Erklärungsansätze herangezogen: Der Sportverein habe sich von der solidargemeinschaftlichen Wertgemeinschaft, in der die Mitglieder ihr Handeln an den Werten, Normen und strukturellen Besonderheiten des Vereins(lebens) ausrichten, zu einer modernen Dienstleistungsorganisation gewandelt, in der Kund:innen themenbezogen bestimmte Vereinsleistungen gemäß individueller Kosten-Nutzen-Kalküle auswählen (z. B. Baur & Braun, 2003; Braun, 2014; Nagel, 2006a; Schlesinger & Nagel, 2013; Strob, 1999; Thieme, 2017; Zimmer, 2007; Zimmer, Basic, & Hallmann, 2011). Dadurch würden sich traditionelle und wertrationale Mitgliedschaftsbeziehungen zugunsten zweckrationaler Beziehungen auflösen. Zweckorientierte Mitglieder würden „ihre Investitionen zeitlicher und finanzieller Ressourcen in Sportvereine nur solange aufrecht[erhalten], wie der in Aussicht gestellte Nutzen einer Vereinsmitgliedschaft – im Sinne der Befriedigung ihrer sportbezogenen und sozialen Interessen – die anfallenden Kosten für die Ressourcenabgabe übersteigt“ (Schlesinger & Nagel, 2013, S. 90; vgl. auch Arbeiten zur sogenannten „Match-Qualität“ von Behrens, Emrich, Hämmerle, & Pierdzioch, 2017).

Neben veränderten individuellen Anreiz- und Erwartungsstrukturen werden auch organisationale Einflussfaktoren diskutiert. Intraorganisationale Veränderungsprozesse wie Verberuflichung oder Öffnung für Nichtmitglieder könnten Ziel-Interessen-Divergenzen zwischen Mitgliedern und Verein befördern und zu vermehrten Vereinsaustritten oder zum Rückzug aus Vereinsaufgaben und -ämtern führen (Klenk, 2011).

Mit seiner Metapher vom „Bowling Alone“ hat Putnam (1995) vor rund einem Vierteljahrhundert der mittlerweile weit verbreiteten Sorge vor rückläufigen Mitgliedschafts- und Engagementquoten ebenso wie abnehmenden expressiven Bindungen an lokale freiwillige Vereinigungen und deren Folgen für den sozialen Zusammenhalt einen sinnfälligen Ausdruck verliehen. Putnams Konzept des Sozialkapitals, welches sowohl auf die klassische Demokratietheorie als auch auf transaktionskostenökonomische Ansätze rekurriert (Braun, 2003), beinhaltet drei wesentliche Elemente: (1) soziales Vertrauen, das die Kooperation zwischen den Akteuren vereinfache, was wiederum für die soziale Koordination unabdingbar sei; (2) die Norm generalisierter Reziprozität, die zur Lösung sozialer Dilemmata beitrage und schließlich (3) Netzwerke zivilgesellschaftlichen Engagements, die entsprechende Formen von Vertrauen entwickeln und generalisierte Reziprozitätsnormen aufrechterhalten würden (Putnam, 1993, S. 170 ff., 1995, S. 67).

Putnam (2000) unterscheidet ferner zwischen Bindungs- und Brückenkapital. Ersteres basiert auf der Entwicklung neuer und Aufrechterhaltung bestehender Beziehungen innerhalb von Gruppen von Menschen mit einem ähnlichen sozialen und kulturellen Hintergrund und ähnlicher Einstellung. In diesen eher geschlossenen Gruppen werde sozialer Zusammenhalt und gegenseitige Unterstützung gefördert, könne es aber auch zu sozialer Stratifikation und Ausgrenzung anderer kommen (Putnam, 2000; Schüttoff, Pawlowski, Downward, & Lechner, 2018). Brückenkapital umfasst ein größeres Bewusstsein und besseres Verständnis für andere und führe zu sozialen Interaktionen und Beziehungen zwischen Individuen mit unterschiedlichen Erfahrungshintergründen und sozialen Herkünften. Es verringere mögliche Konflikte zwischen heterogenen Gruppen und fördere deren Kooperation.

Mit der Corona-Pandemie haben Kontaktbeschränkungen, die in Deutschland eingeführt wurden, um der Ausbreitung des Coronavirus entgegenzuwirken, zu einer grundlegenden „Unterbrechung von Routinen und vertrauten Abläufen in allen gesellschaftlichen Bereichen“ (Beck, 2020, S. 451) beigetragen. Für den verbandlich organisierten Vereinssport kommt erschwerend hinzu, dass durch die Kontaktbeschränkungen sowohl das *Kerngeschäft* des Trainings- und Wettkampfbetriebs als auch das Vereinsleben substanziell eingeschränkt wurden und weitreichend limitiert bleiben. Die Mitgliederbestandsdaten des DOSB weisen für 2020 einen Mitgliederschwund von ca. 3,5 % aus (Rump, 2021). Daten einer repräsentativen Bevölkerungsbefragung bei Erwachsenen kommen zu Mitgliederverlusten von 8 % bei Sportvereinen, 11 % bei anderen binnenorientierten Vereinigungen (z. B. Gesangsverein, Hobbyverein) und 7 % bei außenorientierten Vereinigungen (z. B. Gewerkschaften, Parteien; Braun, Burrmann, & Sielschott, 2021).^1^ Befunde zu möglichen Auswirkungen der Corona-Pandemie auf Engagementquoten und Mitgliederbindung liegen unseres Wissens (noch) nicht vor.

Angesichts der komplexen Einschränkungen der Sport- und geselligen Aktivitäten in den Sportvereinen während des zweiten Lockdowns stellt sich die Frage, wie diese außergewöhnliche soziale Situation die Mitgliederbindung in Sportvereinen beeinflusst hat und welche Bedeutung dem Sozialkapital dabei zukommt.

## Bisheriger Forschungsstand zur Bedeutung von Sozialkapital für die Mitgliederbindung in Sportvereinen

Sportvereine als freiwillige Vereinigungen bilden einen frei gewählten Zusammenschluss von natürlichen Personen, die eine freiwillige Mitgliedschaft in einer formalen Organisationsstruktur eingehen, um ihre jeweils spezifischen Ziele zu verfolgen (Horch, 1992). Die nutzen- bzw. zweckorientierte Mitgliedschaftsbeziehung besteht darin, dass Personen einem Sportverein beitreten und in ihm verbleiben, weil die Vereinsziele mit ihren eigenen Interessen, die sie mit dem vereinsorganisierten Sport verknüpfen, weitgehend übereinstimmen. Mitgliedschaften können aber auch wertorientiert motiviert sein, insofern

als die Mitglieder aus der Überzeugung heraus agieren, dass der Sport im Allgemeinen etwas Wertvolles sei und es folglich Sinn mache, sich für jene Werte einzusetzen, die der Sport explizit oder implizit vermitteln könne – z. B. Fairness und Kameradschaftlichkeit, Leistungsprinzip und Konkurrenz unter gleichen „Startbedingungen“, aber auch „sportliche Lebensführung“ im Sinne eines „ganzheitlichen Lebens“ (Baur, Burrmann, & Nagel, 2003a, S. 163).

Starke und dauerhafte Mitgliederbindungen dürften sich insbesondere dann entwickeln, wenn sich die Mitglieder aufgrund ihrer gemeinsam geteilten – z. B. sportlichen und/oder sozialen – Interessen und Wertorientierungen den anderen Mitgliedern auch affektiv verbunden und in den Verein eingebunden fühlen (Strob, 1999).

Die Entscheidung, Mitglied in einem Sportverein zu bleiben, dürfte demzufolge nicht nur von zweck-, sondern auch von wertrationalen Handlungsorientierungen abhängen. Nagel (2006b) greift deshalb in seinen Analysen der Mitgliederbindung das Essersche Konzept des Framings auf, welches *normatives* und *rationales* Handeln und Entscheiden integriert und zugleich formalisiert. Demnach steige die Wahrscheinlichkeit, dauerhaft Mitglied im Verein zu sein, mit der Zufriedenheit des Mitglieds mit den Leistungen des Vereins sowie der Identifikation und Verbundenheit mit dem Verein. Je größer allerdings wahrgenommene Ziel-Interessen-Divergenzen seien, je kleiner der Nutzen der Vereinsmitgliedschaft und je größer der Nutzen einer möglichen Alternative eingeschätzt werde, desto eher werde die Reflexionsschwelle überschritten und damit über einen Austritt aus dem Verein nachgedacht. Empirisch konnte Nagel (2006b) einen positiven Zusammenhang zwischen der Mitgliederbindung in Sportvereinen und der solidargemeinschaftlichen Handlungsorientierung bzw. der Zufriedenheit ermitteln. Zudem traten jüngere Vereinsmitglieder eher aus dem Verein aus als ältere Mitglieder. In Analysen von Baur et al. (2003a) variierte die Bindung der Mitglieder an ihren Sportverein in Abhängigkeit von Alter, Bildung und sozioökonomischem Status, v. a. aber auch von der Dauer der Mitgliedschaft. Zudem zeigen Mehrebenenanalysen, inwieweit kontextuelle Bedingungen individuelle Entscheidungen und Verhaltensweisen strukturieren und beeinflussen. Unter Kontrolle potenzieller Kontextunterschiede zeigte sich ein signifikanter Einfluss von vier einbezogenen Variablen auf der Individualebene: Die Verbundenheit mit dem Verein, ein als positiv wahrgenommenes soziales Miteinander, die (globale) Zufriedenheit mit dem Verein sowie ein ehrenamtliches Engagement reduzierten das Austrittsrisiko als operationalisiertes Maß für die Mitgliederbindung. Sozialstrukturelle Merkmale wie Alter, Geschlecht, Wettkampfaktivität, Mitgliedschaftsdauer sowie Kinder im Verein hatten keinen signifikanten Einfluss auf das Austrittsrisiko. Zudem veränderten die einbezogenen Kontextfaktoren die Ergebnisse nur geringfügig, was auf einen robusten Einfluss der individuellen Merkmale hindeutet (Schlesinger & Nagel, 2013, 2015).^2^

Einige der genannten mitgliedschaftsbezogenen Faktoren (Verbundenheit mit dem Verein, positiv wahrgenommenes soziales Miteinander, freiwilliges Engagement) lassen sich als Indikatoren von Sozialkapital fassen. So gehen Vertreter:innen der Sozialkapitalforschung davon aus, dass man sich v. a. in der aktiven Mitgliedschafts- und speziell auch Engagementrolle in lokalen Vergemeinschaftungen, die sich durch vielfältige Face-to-face-Interaktionen und emotionale Beziehungen zwischen den Mitgliedern auszeichnen, jene sozialen und politischen Dispositionen und Kompetenzen aneigne, die auch außerhalb der Freiwilligenorganisationen soziales Vertrauen und Reziprozitätsnormen der Akteure erhöhten (z. B. Burrmann, Braun, & Mutz, 2020; Van der Meer & van Ingen, 2009). In verschiedenen Studien vor der Corona-Pandemie konnte gezeigt werden, dass Sozialkapitalindikatoren, wie z. B. Vertrauen bei Vereinsmitgliedern und insbesondere Ehrenamtlichen, höher ausgeprägt sind als bei passiven Vereinsmitgliedern und Nichtmitgliedern (Burrmann, Braun, & Mutz, 2019, 2020; Coffé & Geys, 2007; Mutz & Nobis, 2012; Paxton, 2007; Quintelier, 2008; Seippel, 2006; Stolle, 1998; Van der Meer & van Ingen, 2009; Wollebæk & Strømsnes, 2008), wenngleich die Bedeutung einer aktiven Vereinsmitgliedschaft und auch die Wirkrichtung noch nicht abschließend geklärt sind (Elmose-Østerlund & van der Roest, 2017). Insofern könnte das im (und auch außerhalb) des Sportvereins und anderer freiwilliger Vereinigungen generierte Sozialkapital dazu beitragen, dass Vereinsmitglieder Resilienzen aufbauen und soziale Krisen und Katastrophen oder gegenwärtig die Folgen der Corona-Pandemie besser verkraften als Nichtmitglieder. Ergebnisse einer Bertelsmann-Studie (2020) deuten darauf hin, dass Personen, die einen stärkeren gesellschaftlichen Zusammenhalt erlebten, eine größere psychische Belastbarkeit bzw. Resilienz in der Corona-Pandemie aufweisen. Kwon und Seo (2021) ermittelten auf der Grundlage einer Querschnittstudie im Mai 2020 in Korea, dass das in einem vom Arbeitgeber gesponserten Sportprogramm generierte überbrückende Sozialkapital die schädlichen Auswirkungen der Arbeitsplatzunsicherheit auf das Wohlbefinden der Arbeitnehmer:innen wahrscheinlich abfedern kann.

Die unterschiedlichen Wirkweisen von Sozialkapital zeigten sich auch während und nach einer Katastrophe. Enge Beziehungen zu Freunden und zur Familie unterstützten z. B. nach einer Flutkatastrophe die gemeinsame Nutzung von Essensvorräten, die Befriedigung unmittelbarer Bedürfnisse und den Austausch von Informationen, während Brückenkapital den Zugang zu verschiedenen Ressourcen (z. B. Kontakt zu Hilfsorganisationen) ermöglichte (Meyer, 2017). Gleichwohl berichten Aldrich und Meyer (2015) über verschiedene Studien, in denen hohes Bindungskapital zur Diskriminierung von Personengruppen nach einer Katastrophe geführt hat. Zugleich haben sozioökonomischer Status, Geschlecht, Migrationshintergrund und Behinderung einen Einfluss darauf, wie die Akteur:innen während einer Katastrophe auf Sozialkapital zugreifen und es nutzen (Meyer, 2017). So waren Personen mit niedrigerem Einkommen und Migrationshintergrund stärker auf Unterstützung durch Freunde und Familie angewiesen, während der Zugang zu überbrückenden Netzwerken – das könnten auch Sportvereine oder andere freiwillige Vereinigungen sein – erschwert war (Brouwer & Nhassengo, 2006; Elliott, Haney, & Sams-Abiodun, 2010; Hawkins & Maurer, 2010). Insbesondere Singles und Alleinerziehende, Befragte mit Migrationshintergrund, Personen mit geringem Einkommen und/oder niedriger Bildung sowie Menschen mit körperlicher Beeinträchtigung oder Erkrankung nahmen den gesellschaftlichen Zusammenhalt während der Corona-Pandemie als vergleichsweise schwach wahr (Bertelsmann-Stiftung, 2020).

Überdies traten während der Corona-Pandemie andere Mitgliedergruppen aus den Sportvereinen aus als vor Beginn der Pandemie. Die Unterschiede ließen sich nicht allein auf unerwartete strukturelle Veränderungen in der persönlichen Lebenssituation (z. B. Kurzarbeit, mangelnde externe Ressourcenmobilisierung) zurückführen. Auffällig war, dass die Austritte bei den Sportvereinen vor allem auch zuungunsten jener gesellschaftlichen Gruppen ausfielen, für die die Sportvereinsforschung seit Langem schon über ungünstigere Zugangschancen zu Sportvereinen diskutiert, nämlich Personen mit Migrationshintergrund, bildungsfernere Gruppen, aber auch Frauen und Eltern mit jüngeren Kindern (Braun et al., 2021). Bekannte soziale Ungleichheitsrelationen im Hinblick sowohl auf die Nutzung von Sozialkapital als auch auf Partizipation in (Sport‑)Vereinen scheinen sich während der Pandemie verschärft zu haben. Die Ergebnisse könnten aber auch darauf hindeuten, dass Mitgliedschaften eines bestimmten Umfangs an sozialen Ressourcen im persönlichen Lebensumfeld bedürfen, um sich einem Sportverein dauerhaft anzuschließen.

Alles in allem ist der bisherige Forschungsstand zur Relevanz von Sozialkapitalindikatoren für die dauerhafte Mitgliedschaft in Freiwilligenvereinigungen während und nach einer Katastrophe oder speziell auch während der Corona-Pandemie noch recht schmal (Albrecht, 2018; Aldrich & Meyer, 2015; Meyer, 2017; Nakagawa & Shaw, 2004). Eine Ausnahme bildet die Untersuchung von Lee und Fraser (2019): Rund ein Jahr nach der Nuklearkatastrophe in Fukushima waren japanische Befragte, die Erfahrungen mit Naturkatastrophen hatten, häufiger Mitglieder und aktiv engagiert in werteorientierten Freiwilligenorganisationen als Befragte ohne entsprechende Erfahrungen. Der vorliegende Beitrag greift diese Forschungslücke auf, indem die Mitgliederbindung in Sportvereinen nach der Corona-Pandemie abgeschätzt werden soll, wobei v. a. die Relevanz von verschiedenen Sozialkapitalindikatoren betrachtet wird.

Auf der Grundlage des bisherigen Forschungsstandes wird *erstens* von der Annahme ausgegangen, dass die Ausprägung von Sozialkapital wie z. B. Vertrauen oder wahrgenommene Reziprozität unter den Mitgliedern die Wahrscheinlichkeit erhöht, auch nach der Corona-Pandemie Mitglied im Sportverein zu sein. Die Mitgliederbindung dürfte aber *zweitens *auch durch mitgliedschaftsbezogene Merkmale wie der Zufriedenheit der Mitglieder mit dem Krisenmanagement des Vereins oder mit den wahrgenommenen Corona-bedingten Einschränkungen im Hinblick auf Vereinsangebote und -aktivitäten vorhergesagt werden. Als Kontrollvariablen werden soziodemographische Merkmale in die Analysen einbezogen.

## Methode

Basis der folgenden empirischen Analysen ist eine Bevölkerungsbefragung, die von Kantar Public (Deutschland, Standort München) im Zeitraum vom 14.12.2020 bis zum 06.01.2021 als repräsentative Online-Befragung (CAWI) durchgeführt wurde.^3^

Die Befragten wurden im Rahmen des konzerninternen Online-Panels (233.000 Adressen, bundesweit breit gestreut, Lightspeed GMI) rekrutiert. Die Stichprobe der Befragung umfasst 3247 Personen ab 18 Jahren mit einer Quotierung nach Alter, Geschlecht, Schulbildung und Region. Zum Ausgleich von Stichprobenverzerrungen im Vergleich zur Grundgesamtheit wurden die Daten gewichtet, indem soziodemographische Merkmale an entsprechende Daten der amtlichen Statistik angepasst worden sind. Die Effektivität der Gewichtung liegt bei ca. 82 %.

Um eine Vergleichbarkeit zu anderen Studien herzustellen, wurde auf bereits geprüfte Items und Skalen aus empirischen Studien der Engagement- und Sportvereinsforschung zurückgegriffen. Teilweise wurden Items modifiziert, um die aktuelle Corona-Pandemie abbilden zu können. Die Werte für die interne Konsistenz der Kurzskalen liegen mit einer Ausnahme – dem Ingroup-Vertrauen – zwischen α = 0,61 und 0,81 und weisen damit akzeptable bis zufriedenstellende Werte auf (Schmitt, 1996).

### Angaben zur Stichprobe

Für die Analysen werden 591 Personen einbezogen, die zum Zeitpunkt der Befragung in mindestens einem Sportverein Mitglied waren (Tab. 1). Dabei ist zu berücksichtigen, dass die Zusammensetzung der Teilstichprobe der in der repräsentativen Bevölkerungsstichprobe enthaltenen Sportvereinsmitglieder nicht der typischen Zusammensetzung der erwachsenen Mitglieder in Sportvereinen entsprechen muss. Die Teilstichprobe kann verzerrt sein, wenngleich sich die in Sportvereinen typische Überrepräsentation von Männern, jüngeren Mitgliedern, Abiturient:innen und Mitgliedern aus den alten Bundesländern auch in der Teilstichprobe wiederfindet. Die Berechnungen werden mit der ungewichteten Teilstichprobe der Sportvereinsmitglieder durchgeführt, wobei in den Regressionsmodellen soziodemographische Merkmale kontrolliert werden.^4^

**Tab. 1:** Stichprobenbeschreibung

	Alle Befragten			Sportvereinsmitglieder		
	Ungewichtet		Gewichtet	Ungewichtet		Gewichtet
	*N*	%	%	*N*	%	%
*Geschlecht*						
Männlich	1548	48	49	348	59	60
Weiblich	1695	52	51	243	41	40
Divers	4	0,1	–	0	–	–
*Alter*						
18 bis 40 Jahre	992	31	32	226	38	42
Ab 41 Jahre	2255	69	68	365	62	58
*Bildung*						
Kein Abitur	2092	66	65	287	49	49
Mit Abitur	1108	34	35	300	51	51
*Wohnort*						
Unter 20.000 EW	1399	43	41	273	46	43
Ab 20.000 EW	1847	57	59	318	54	57
*Region*						
Ost	638	20	20	86	15	13
West	2609	80	80	505	85	87

*EW* Einwohner:innen

### Abhängige Variable

Die Mitgliederbindung wurde mit Antwortkategorien von (1) „Stimme überhaupt nicht zu“ bis (4) „Stimme voll und ganz zu“ über das Item erfasst: „Ich werde auch nach der Corona-Pandemie noch Mitglied in diesem Verein sein.“

### Unabhängige Variablen – Indikatoren für Sozialkapital

In Anlehnung an Stolle (2001) wird in unserem Beitrag ein breiteres Verständnis von Sozialkapital angelegt, das auch verschiedene individuelle Einstellungs- und Verhaltensindikatoren umfasst. „They all have in common that they tap a certain sense of engagement, a readiness to cooperate, to give the benefit of the doubt, to commit, to get involved, and to trust“ (Stolle, 2001, S. 210).

Dabei werden drei Indikatoren – freiwilliges Engagement, Reziprozitätsnormen und Mitgliedervertrauen – herangezogen, die im Sportverein generiert werden. Vier weitere Indikatoren – Ingroup-Vertrauen, Outgroup-Vertrauen, gesellige Orientierungen und Hilfsbereitschaft – dürften v. a. außerhalb des Sportvereins ausgebildet werden.

Für das freiwillige Engagement im Sportverein wurde eine Dummy-Variable gebildet (Eigenkonstruktion). Die Befragten mussten mindestens einem von zwei Items („Ich helfe regelmäßig mit“, „Ich übe im Verein ein Amt aus“) zustimmen, um als „freiwillig engagiert“ kategorisiert zu werden.

Die Skala zu den Reziprozitätsnormen bezieht sich v. a. auf den Grad der interaktiven Konnektivität und sozialen Nähe unter den Mitgliedern. Beispiel-Items hierfür sind: „Viele Vereinsmitglieder haben während der Corona-Pandemie ein offenes Ohr für Sorgen und Probleme anderer Mitglieder“, „Von den anderen Vereinsmitgliedern werde ich während der Corona-Pandemie häufig um Rat gefragt“, „In meinem Verein kann ich mich gerade in Zeiten der Corona-Pandemie so geben, wie ich bin“ (Eigenkonstruktion; 6 Items, Cronbachs Alpha = 0,81, Antwortkategorien von (1) „Stimme überhaupt nicht zu“ bis (4) „Stimme voll und ganz zu“).

Ein Item bezieht sich auf das Vertrauen zu den anderen Mitgliedern des Vereins mit Antwortkategorien von (1) „Vertraue gar nicht“ bis (4) „Vertraue völlig“.

Ingroup-Vertrauen umfasst das soziale Vertrauen im Nahbereich. Erfasst wird, inwieweit die Befragten Mitgliedern ihrer Familie, Menschen in ihrer Nachbarschaft und Menschen, die sie persönlich kennen, vertrauen (Delhey, Newton, & Welzel, 2011; 3 Items, Cronbachs Alpha = 0,56, Antwortkategorien von (1) „Vertraue gar nicht“ bis (4) „Vertraue völlig“).

Mit dem Out-Group-Vertrauen wird das soziale Vertrauen außerhalb des Nahbereichs erfasst. Hier wird ermittelt, inwieweit die Befragten Menschen, denen sie zum ersten Mal begegnen, Menschen mit einer anderen Religion und Menschen mit anderer Nationalität, vertrauen (Delhey et al., 2011; 3 Items, Cronbachs Alpha = 0,81, Antwortkategorien von (1) „Vertraue gar nicht“ bis (4) „Vertraue völlig“).

Gesellige Orientierungen thematisieren das soziale Netzwerk einer Person und die Bedeutung, die Freunden und Bekannten beigemessen wird. Beispiel-Items hierfür sind: „Es fällt mir schwer, Freundschaften zu schließen“ (invertiert), „Ich feiere meinen Geburtstag gerne mit vielen Leuten“ (Vester, von Oertzen, Geiling, Hermann, & Müller, 2001; 6 Items, Cronbachs Alpha = 0,80, Antwortkategorien von (1) „Trifft überhaupt nicht zu“ bis (4) „Trifft ganz genau zu“).

Hilfsbereitschaft bezieht sich auf die Bereitschaft einer Person, anderen zu helfen und sich für andere einzusetzen. Beispiel-Items hierfür sind: „Ich helfe gerne anderen Leuten, wenn ich dazu in der Lage bin“, „Ich bin jederzeit bereit, mich für die Interessen anderer einzusetzen, wenn mich das überzeugt“ (Vester et al., 2001; 4 Items, Cronbachs Alpha = 0,69, Antwortkategorien von (1) „Trifft überhaupt nicht zu“ bis (4) „Trifft ganz genau zu“).

### Unabhängige Variablen – mitgliedschaftsbezogene Merkmale

Die Zufriedenheit mit dem Krisenmanagement des Vereins wurde mit dem Item erhoben: „Mit dem Krisenmanagement meines Vereins während der Corona-Pandemie bin ich sehr zufrieden“ (Eigenkonstruktion; Antwortkategorien von (1) „Stimme überhaupt nicht zu“ bis (4) „Stimme voll und ganz zu“).

Das Ausmaß von Corona-bedingten Einschränkungen in der Angebotsstruktur der Sportvereine wurde auf der Grundlage von drei Items (Eigenkonstruktion) ermittelt. Die Sportvereinsmitglieder sollten angeben, inwieweit (1) Angebote in ihrem Verein, (2) gesellige Aktivitäten des Vereins und (3) private Kontakte zu anderen Vereinsmitgliedern durch Corona seltener, unverändert oder häufiger stattfanden. Dabei sollten auch (neue) digitale Angebote berücksichtigt werden. Die Aktivitäten wurden aufsummiert und für die Analysen Dummy-codiert: 1 = in mindestens zwei Bereichen fanden Aktivitäten seltener statt, 0 = in maximal einem Bereich fanden Aktivitäten seltener statt.

Die Skala Konflikte wird auf der Grundlage der zwei Items „In meinem Verein häufen sich seit der Corona-Pandemie die Konflikte“ und „Viele Vereinsmitglieder sind seit der Corona-Pandemie nicht mehr auf meiner Wellenlänge“ gebildet (Eigenkonstruktion; 2 Items, Cronbachs Alpha = 0,71, Antwortkategorien von (1) „Stimme überhaupt nicht zu“ bis (4) „Stimme voll und ganz zu“).

Erfragt wurde die Mitgliedschaftsdauer, wobei die Kategorien „unter einem Jahr“, „1 bis unter 3 Jahre“, „3 bis 10 Jahre“ zusammengefasst wurden, sodass differenziert wird zwischen Personen, die seit höchstens 10 Jahren Vereinsmitglied sind und jenen, die mehr als 10 Jahre Mitglied sind.

### Kontrollvariablen

Als soziodemographische Merkmale wurden in die Analysen einbezogen: Alter, Geschlecht (0 = weiblich vs. 1 = männlich),^5^ Bildungsniveau (0 = Abitur vs. 1 = kein Abitur), Wohnort ab 20.000 Einwohner:innen (0 = ja vs. 1 = nein), Migrationsstatus, also Befragte, die selbst und/oder deren Elternteile nicht in Deutschland geboren sind (0 = ja vs. 1 = nein), Einkommen unter 2500 € Nettoeinkommen im Haushalt (0 = ja vs. 1 = nein), Kinder bis 13 Jahre im Haushalt (0 = ja vs. 1 = nein).

## Empirische Befunde

Die Mitgliederbindung in Sportvereinen ist nach wie vor hoch: 68 % der Sportvereinsmitglieder stimmen der Aussage voll und ganz zu, dass sie auch nach der Corona-Pandemie noch Mitglied in diesem Verein sein werden. Die Bindung und die Zufriedenheit mit dem Krisenmanagement des Vereins sind besonders bei Älteren und bei alteingesessenen Mitgliedern hoch.^6^ Die Unterschiede bleiben auch nach der Alphafehleradjustierung mittels Bonferroni-Korrektur signifikant (Tab. 2).

**Tab. 2:** Sozialkapitalindikatoren, Mitgliederbindung und Zufriedenheit mit dem Krisenmanagement differenziert nach soziodemographischen und vereinsstrukturellen Merkmalen

	Mitgliederbindung		Krisenmanagement		Einschränkungen Angebote		Konflikte		Reziprozität		Mitgliedervertrauen	
	M	SD	M	SD	M	SD	M	SD	M	SD	M	SD
*Geschlecht*												
Männlich	3,61	0,64	3,13	0,74	0,75	0,43	2,06	0,81	2,76	0,60	2,99	0,52
Weiblich	3,61	0,64	3,10	0,76	0,87	0,33	1,86	0,76	2,54	0,68	2,82	0,66
p(*t-*Test)					***		**		***		**	
*Alter*												
18 bis 39 Jahre	3,44	0,71	2,95	0,77	0,69	0,46	2,23	0,85	2,73	0,59	2,86	0,66
Ab 40 Jahre	3,71	0,56	3,23	0,71	0,87	0,33	1,82	0,72	2,63	0,67	2,97	0,53
p(*t*-Test)	***		***		***		***				*	
*Bildung*												
Abitur	3,58	0,64	3,11	0,71	0,75	0,43	2,10	0,83	2,70	0,64	2,92	0,63
Kein Abitur	3,64	0,64	3,13	0,78	0,85	0,36	1,86	0,73	2,64	0,65	2,93	0,55
p(*t*-Test)					**		***					
*Einkommen*												
Unter 2500 €	3,52	0,70	3,01	0,80	0,76	0,43	2,01	0,82	2,64	0,68	2,93	0,67
ab 2500 €	3,66	0,59	3,18	0,71	0,83	0,38	1,96	0,78	2,69	0,63	2,92	0,53
p(*t*-Test)	*		*		*							
*Migration*												
Mit	3,41	0,71	3,05	0,81	0,70	0,46	2,15	0,87	2,66	0,75	2,87	0,63
Ohne	3,64	0,62	3,13	0,74	0,81	0,39	1,96	0,78	2,67	0,63	2,93	0,58
p(*t*-Test)	*											
*Wohnort*												
ab 20.000 EW	3,63	0,59	3,11	0,69	0,76	0,43	2,07	0,83	2,70	0,66	2,93	0,60
< 20.000 EW	3,58	0,69	3,13	0,81	0,85	0,36	1,87	0,74	2,63	0,63	2,92	0,58
p(*t*-Test)					**		**					
*Kinder <* *13* *Jahre*												
Ja	3,54	0,69	3,01	0,76	0,73	0,44	2,11	0,80	2,76	0,60	2,89	0,57
Nein	3,63	0,62	3,15	0,74	0,82	0,38	1,94	0,79	2,65	0,66	2,94	0,59
p(*t*-Test)					*		*					
*Mitgliedschaftsdauer*												
Bis 10 Jahre	3,46	0,72	2,98	0,79	0,73	0,44	2,17	0,85	2,62	0,71	2,85	0,68
Mehr als 10 Jahre	3,75	0,49	3,25	0,68	0,86***	0,34	1,82	0,70	2,73	0,57	2,99	0,48
*p*(*t*-Test)	***		***				***		*		**	
*Einschränkungen Angebote*												
2 Bereiche	3,35	0,77	3,07	0,78	–	–	2,41	0,91	2,91	0,64	3,00	0,63
2–3 Bereiche	3,68	0,58	3,13	0,74	–	–	1,89	0,72	2,66	0,59	2,91	0,57
p(*t*-Test)	***						***		***			

*EW* Einwohner:innen

Signifikanzniveau: ***p < 0,001, **p < 0,01, *p < 0,05 ohne Bonferroni-Korrektur

Außerdem variieren die Corona-bedingten Einschränkungen des Sportvereinsbetriebs signifikant mit soziodemographischen Merkmalen der Mitglieder: Frauen, Ältere und alteingesessene Mitglieder geben häufiger an, dass sportliche und soziale Vereinsaktivitäten während der Corona-Pandemie seltener stattgefunden haben als Männer, Jüngere und Mitglieder mit kürzerer Vereinszugehörigkeit. Starke Corona-bedingte Einschränkungen des Vereinsbetriebs gehen zudem mit einem geringeren Grad an interaktiver Konnektivität und Reziprozität unter den Mitgliedern und einem geringeren Outgroup-Vertrauen, zugleich aber auch mit weniger Konfliktpotenzial unter den Mitgliedern und einer stärkeren Mitgliederbindung einher.

Sozialkapitalindikatoren differenzieren v. a. nach Geschlecht, Alter und Einkommen der Mitglieder. Während der Corona-Pandemie nehmen weibliche im Vergleich zu männlichen Sportvereinsmitgliedern eine signifikant geringere Ausprägung an interaktiver Konnektivität und sozialer Nähe unter den Mitgliedern wahr. Erstere engagieren sich häufiger freiwillig (66 % zu 44 %, t(536,07) = −5,51; *p* < 0,001), wenngleich sie seltener als Männer ein Ehrenamt ausüben (11 % zu 25 %, t(588,69) = 4,68; *p* < 0,001). Zudem ist bei Frauen das soziale Vertrauen außerhalb des Nahbereichs geringer ausgeprägt als bei Männern (Tab. 3). Ältere Mitglieder engagieren sich häufiger freiwillig im Verein und weisen im Vergleich zu Jüngeren höhere Werte beim sozialen Vertrauen im Nahraum und bei der Hilfsbereitschaft auf. Und schließlich kann bei den Mitgliedern mit höherem Einkommen ein höheres Ingroup-Vertrauen ermittelt werden als bei Geringverdienern. Die berichteten Unterschiede bleiben auch nach Alphafehleradjustierung signifikant, berücksichtigen aber keine (möglichen) relevanten Drittvariablen.

**Tab. 3:** Sozialkapitalindikatoren, Mitgliederbindung und Zufriedenheit mit dem Krisenmanagement differenziert nach soziodemographischen und vereinsstrukturellen Merkmalen

	Freiwilliges Engagement		Ingroup-Vertrauen		Outgroup-Vertrauen		Geselligkeit		Hilfsbereitschaft	
	M	SD	M	SD	M	SD	M	SD	M	SD
*Geschlecht*										
Männlich	0,44	0,50	3,15	0,45	2,40	0,69	2,69	0,54	3,15	0,44
Weiblich	0,66	0,47	3,07	0,48	2,21	0,57	2,57	0,54	3,18	0,43
p(*t*-Test)	***		*		***		**			
*Alter*										
18 bis 39 Jahre	0,42	0,50	3,00	0,49	2,36	0,71	2,67	0,52	3,08	0,44
Ab 40 Jahre	0,60	0,49	3,19	0,43	2,30	0,61	2,62	0,56	3,21	0,43
p(*t*-Test)	***		***						***	
*Bildung*										
Abitur	0,47	0,50	3,12	0,49	2,40	0,68	2,66	0,54	3,15	0,46
Kein Abitur	0,60	0,49	3,12	0,44	2,23	0,60	2,61	0,54	3,17	0,41
p(*t*-Test)	**				**					
*Einkommen*										
Unter 2500 €	0,56	0,50	3,03	0,53	2,25	0,68	2,57	0,58	3,11	0,43
Ab 2500 €	0,52	0,50	3,17	0,41	2,37	0,63	2,68	0,52	3,19	0,44
p(*t*-Test)			***		*		*		*	
*Migrationsstatus*										
Mit	0,44	0,50	3,04	0,58	2,29	0,69	2,57	0,52	3,16	0,46
Ohne	0,54	0,50	3,12	0,45	2,32	0,65	2,65	0,55	3,16	0,43
p(*t*-Test)										
*Wohnort*										
Ab 20.000 EW	0,52	0,50	3,11	0,46	2,37	0,64	2,68	0,55	3,16	0,43
< 20.000 EW	0,55	0,50	3,13	0,49	2,26	0,66	2,60	0,53	3,16	0,44
p(*t*-Test)										
*Kinder <* *13 Jahre*										
Ja	0,45	0,50	3,11	0,47	2,39	0,65	2,72	0,50	3,19	0,47
Nein	0,56	0,50	3,12	0,46	2,30	0,65	2,61	0,55	3,15	0,42
p(*t*-Test)	*						*			
*Mitgliedschaftsdauer*										
Bis 10 Jahre	0,54	0,50	3,05	0,50	2,31	0,70	2,59	0,56	3,10	0,45
Mehr als 10 Jahre	0,52	0,50	3,18	0,43	2,34	0,69	2,68	0,53	3,21	0,42
p(*t*-Test)			**				*		**	
*Einschränkungen Angebote*										
< 2 Bereiche	0,41	0,50	3,04	0,49	2,61	0,66	2,80	0,54	3,04	0,43
2–3 Bereiche	0,54	0,50	3,14	0,45	2,26	0,62	2,62	0,53	3,19	0,43
p(*t*-Test)	**		*		***		**		**	

*EW* Einwohner:innen

Signifikanzniveau: ***p < 0,001, **p < 0,01, *p < 0,05 ohne Bonferroni-Korrektur

Um die Annahmen zu prüfen, inwieweit Sozialkapitalindikatoren und mitgliedschaftsbezogene Merkmale die Mitgliederbindung vorhersagen, werden zunächst separate Regressionsmodelle für die Kontrollvariablen (Modell 1), Sozialkapitalindikatoren (Modell 2a, b) und mitgliedschaftsbezogenen Merkmale (Modell 3a, b) berechnet. Das vierte Modell umfasst dann alle Prädiktoren. Zuvor wurden entsprechende Voraussetzungen geprüft und u. a. Multikollinearitätsanalysen durchgeführt. Die höchsten bivariaten Zusammenhänge zeigen sich zwischen Reziprozitätsnormen und Geselligkeitsorientierung sowie zwischen Mitgliedervertrauen und Ingroup-Vertrauen (jeweils r = 0,47; *p* < 0,001; Tab. 6 im Anhang). Die Werte für die Toleranz und Varianzinflationsfaktoren bleiben ebenfalls unter den kritischen Werten von TOL > 0,25 sowie VIF < 4 (Urban & Mayerl, 2018). Die Parameterschätzungen werden mit robusten Standardfehlern vorgenommen, um Streuungsungleichheiten zu begegnen (HC3, vgl. Hayes & Cai, 2007).

Die separaten Regressionsmodelle lassen Folgendes erkennen (Tab. 4): Die Wahrscheinlichkeit, auch nach der Corona-Pandemie Mitglied im Sportverein zu sein, steigt mit dem Alter und Einkommen der Mitglieder und sinkt tendenziell, wenn Befragte und/oder deren Eltern nicht in Deutschland geboren wurden (Modell 1). Soziodemographische Merkmale tragen mit knapp 7 % zur Varianzaufklärung der Mitgliederbindung bei.

**Tab. 4:** Prädiktoren der Mitgliederbindung in Sportvereinen als abhängige Variablen. Parameterschätzungen mit robusten Standardfehlern. OLS-Regressionen

	Mitgliederbindung in Sportvereinen					
	Modell 1	Modell 2a	Modell 2b	Modell 3a	Modell 3b	Modell 4
Konstante	2,941***	1,647***	1,331***	2,852	2,687***	1,721***
Mitgliedervertrauen	–	0,078	0,073	–	–	0,038
Reziprozität	–	0,117*	0,130*	–	–	0,058
Freiwilliges Engagement	–	−0,013	−0,042	–	–	−0,053
Gesellige Orientierung	–	−0,130*	−0,143*	–	–	−0,108+
Hilfsbereitschaft	–	0,419***	0,394***	–	–	0,358***
Ingroup-Vertrauen	–	0,231**	0,188*	–	–	0,098
Outgroup-Vertrauen	–	−0,121**	−0,125**	–	–	−0,086*
Konflikte im Verein	–	–	–	−0,094**	−0,091*	−0,069+
Zufriedenheit mit Krisenmanagement	–	–	–	0,231***	0,223***	0,149**
Corona-bedingte Einschränkungen Vereinsangebote	–	–	–	0,203**	0,203**	0,152*
Mitgliedschaftsdauer	–	–	–	0,147**	0,114*	0,088
(Kein) Migrationsstatus	0,166+	–	0,188*	–	0,013	0,044
Geschlecht	0,030	–	0,023	–	0,052	0,053
Abitur	−0,039	–	−0,033	–	−0,034	−0,051
Kinder im Haushalt	0,013	–	0,021	–	−0,019	0,011
Alter	0,010***	–	0,007***	–	0,005**	0,004*
Wohnort	−0,057	–	−0,073	–	−0,126*	−0,107*
(Höheres) Einkommen	0,136*	–	0,072	–	0,011	0,010
Korr. R^2^	0,066	0,155	0,195	0,178	0,197	0,255
F	6,842	15,038	10,217	28,423	12,224	10,155

Signifikanzniveau: ***p < 0,001, **p < 0,01, *p < 0,05, +*p* < 0,10

Sozialkapitalindikatoren, die sich eng auf den Sportverein beziehen, tragen weniger zur Vorhersage der Mitgliederbindung bei als Sozialkapitalindikatoren mit stärkerem Bezug auf die Vereinsumwelt (Modell 2a). Neben den im Verein wahrgenommenen Reziprozitätsnormen erhöhen Hilfsbereitschaft und Ingroup-Vertrauen die Bindung an den Verein, während Geselligkeitsorientierung und Outgroup-Vertrauen die Wahrscheinlichkeit verringern, auch nach der Pandemie noch Mitglied im Verein zu sein. Die Modellgüte beträgt ca. 16 %. Nach Einbeziehung soziodemographischer Merkmale bleiben die Zusammenhänge bestehen, die Varianzaufklärung erhöht sich auf knapp 20 % (Modell 2b).

Alle in die Regressionsanalyse aufgenommenen mitgliedschaftsbezogenen Merkmale tragen mit knapp 18 % zur Varianzaufklärung bei (Modell 3a). Die Bindung an den Verein wird wahrscheinlicher, je zufriedener die Mitglieder mit dem Krisenmanagement des Vereins sind, je mehr Corona-bedingte Einschränkungen der Vereinsaktivitäten von den Mitgliedern wahrgenommen werden und je länger eine Vereinsmitgliedschaft bereits besteht. Indes verringern wahrgenommene Konflikte die Wahrscheinlichkeit einer Mitgliederbindung. Auch diese Zusammenhänge bleiben bestehen, wenn soziodemographische Merkmale kontrolliert werden. Die Modellgüte liegt im Modell 3b ebenfalls bei knapp 20 %.

Unter Einbeziehung aller Prädiktoren erhöht sich die Modellgüte auf knapp 26 %. Neben wenigen soziodemographischen Merkmalen tragen v. a. mitgliedschaftsbezogene Merkmale zur Varianzaufklärung bei. Hilfsbereitschaft, Outgroup-Vertrauen und tendenziell auch Geselligkeitsorientierung stellen im Gesamtmodell die einzigen bedeutsamen Sozialkapitalindikatoren dar, wenngleich sie mit beachtlichen Gewichten zur Varianzaufklärung beitragen (Modell 4).

## Diskussion der Ergebnisse

Im Beitrag wurde analysiert, inwieweit Sozialkapitalindikatoren zur Vorhersage der Mitgliederbindung an Sportvereine nach einer Katastrophe – hier nach der Corona-Pandemie – beitragen. Zunächst kann konstatiert werden, dass die Mitgliederbindung nach wie vor hoch ist. Der Anteil an Vereinsmitgliedern, die ihrem Verein die Treue halten wollen, ist ähnlich hoch wie in Erhebungen vor der Corona-Pandemie (Schlesinger & Nagel, 2018). Die Exit-Option scheint nach wie vor nur *eine* – und nicht die wahrscheinlichste – mögliche Handlungsreaktion auf durch Ziel-Interessen-Divergenzen hervorgerufene Unzufriedenheit der Mitglieder mit ihrem Verein zu sein (Klenk, 2011).

Das Austrittsrisiko scheint für vulnerablere Gruppen – wie Personen mit geringem Einkommen und/oder Migrationshintergrund – größer zu sein. Zwar können die empirischen Befunde nicht kausal auf die Corona-Pandemie zurückgeführt werden, Drop-out-Analysen (Braun et al., 2021) lassen die Befunde aber plausibel erscheinen. Allerdings tragen die Merkmale nicht mehr signifikant zur Varianzaufklärung der Mitgliederbindung bei, wenn Sozialkapitalindikatoren und mitgliedschaftsbezogene Merkmale in die Regressionsanalysen einbezogen werden.

Unter Einbeziehung soziodemographischer und mitgliedschaftsbezogener Merkmale tragen insbesondere Hilfsbereitschaft und geringes Outgroup-Vertrauen signifikant zur Vorhersage der Mitgliederbindung bei. Solidargemeinschaftliche Orientierungen – und dazu dürfte auch die Hilfsbereitschaft gehören – waren schon in anderen Erhebungen relevante Prädiktoren der Mitgliederbindung (Nagel, 2006b). Daran scheint die Corona-Pandemie nichts geändert zu haben. Während der Pandemie scheint jedoch das überbrückende Potenzial der Sportvereine gelitten zu haben, denn offensichtlich fühlen sich v. a. Mitglieder dem Verein nicht so stark verbunden, deren Vertrauen sich insbesondere auch auf Personen bezieht, die *fremd* sind, eine andere Religion und/oder Nationalität aufweisen. Inwieweit sich hier – wie bei Aldrich und Meyer (2015) negative Effekte eines hohen sozialen Bindungskapitals andeuten, muss in weiterführenden Trendanalysen geprüft werden.

Freiwilliges Engagement trägt nicht zur Vorhersage der Mitgliederbindung bei. Allerdings war der Forschungsstand zur Bedeutung einer aktiven Vereinsmitgliedschaft im Hinblick auf die Generierung von Sozialkapital bereits vor der Corona-Pandemie inkonsistent (Elmose-Østerlund & van der Roest, 2017). Ein Vorteil unserer Erhebung besteht darin, dass verschiedene Sozialkapitalindikatoren erhoben wurden, die sich dem Bindungskapital (z. B. Ingroup-Vertrauen) oder Brückenkapital (z. B. Outgroup-Vertrauen) zuordnen lassen und eher im Sportverein (z. B. Mitgliedervertrauen) oder auch außerhalb des Sportvereins (z. B. Hilfsbereitschaft) entwickelt werden können. Wie diese Indikatoren zusammenhängen, zu welchem Zweck (z. B. zur sozialen Integration oder zur Erhaltung bestehender Netzwerke) sie genutzt werden und welche Veränderungen sich während der Pandemie ergeben haben, sind weiterführende Forschungsfragen, die u. a. mittels qualitativer Interviews mit Personen aus unterschiedlichen Gruppen (aktuelle Sportvereinsmitglieder, während der Pandemie ausgetretene Sportvereinsmitglieder, Nichtmitglieder) eruiert werden könnten.

Schließlich erweist sich die Zufriedenheit mit dem Krisenmanagement des Vereins im Gesamtmodell – wie bereits in älteren Studien (z. B. Nagel, 2006b; Schlesinger & Nagel, 2013) – als bedeutsamer Prädiktor zur Vorhersage der Mitgliederbindung. Das Austrittsrisiko verringert sich zumindest tendenziell, wenn weniger Konflikte unter den Vereinsmitgliedern existieren (bzw. als solche wahrgenommen werden) und wenn Vereinsaktivitäten während der Corona-Pandemie stark reduziert werden mussten. Letzteres könnte vermutlich darauf zurückzuführen sein, dass von den Einschränkungen v. a. Kontakt- und Mannschaftssportarten betroffen waren sowie Wettkämpfe ausfallen mussten. Sportvereine, die sich auf den Wettkampfsport konzentrieren, scheinen ihre Mitglieder stärker binden zu können als Sportvereine, in denen der Breitensport dominiert (Baur, Burrmann, & Nagel, 2003b).

Nicht alle Einflussfaktoren lassen sich schlüssig interpretieren. So wird in den Modellen 3b und 4 die Wohnortgröße relevant. Ein größerer Wohnort geht erwartungswidrig mit einer stärkeren Mitgliederbindung einher. Da die Nullkorrelationen sehr niedrig ausfallen, ist von Mediationseffekten auszugehen. Alles in allem zeigen die Regressionsanalysen, dass sowohl verschiedene Sozialkapitalindikatoren als auch mitgliedschaftsbezogene Merkmale die Mitgliederbindung vorhersagen können. Damit können beide Annahmen zumindest teilweise bestätigt werden. Mit dem Gesamtmodell wird zudem eine hohe Varianzaufklärung erzielt (Cohen, 1988).

Ferner deuten die Befunde einerseits darauf hin, dass die physischen und sozialen Kontaktbeschränkungen während des zweiten Lockdowns dazu geführt haben könnten, dass in Sportvereinen, in denen sportliche und soziale Vereinsaktivitäten und/oder private Kontakte zu den Mitgliedern seltener stattgefunden haben, auch weniger Sozialkapital – zumindest im Sinne von wahrgenommener Reziprozität und sozialer Nähe unter den Mitgliedern – generiert werden konnte als in Sportvereinen, die Vereinsaktivitäten ggf. auch durch die Einführung digitaler Angebote nicht so stark reduzieren mussten. Andererseits scheinen starke Einschränkungen im Sportvereinsbetrieb nicht unbedingt mit einem Rückgang freiwillig Engagierter einhergegangen zu sein, wenngleich bei den Berechnungen nur der Anteil an freiwillig Engagierten und nicht der entsprechende zeitliche Aufwand berücksichtigt werden konnte. An dieser Stelle muss allerdings auf Grenzen der Querschnittstudie hingewiesen werden. Durch die zeitgleiche Erhebung der unabhängigen und abhängigen Variablen wird eine Wirkrichtung festgelegt, die sich plausibilisieren lässt, aber durch längsschnittliche Daten abgesichert werden muss.

Eine weitere Limitation der vorliegenden Studie betrifft den Umstand, dass die Corona-Pandemie zum Befragungszeitpunkt nicht abgeschlossen war. Wie sich allerdings seit dem Befragungszeitpunkt unter den im Jahr 2021 weiterhin bestehenden Corona-bedingten Einschränkungen des Sportvereinsbetriebs die Mitgliederbindung und Sozialkapitalindikatoren weiterentwickelt haben, werden zukünftige Untersuchungen zeigen müssen. Während einige Forschungsarbeiten nahelegen, dass das überbrückende Sozialkapital nach einer Katastrophe im Laufe der Zeit an Bedeutung gewinnt, deuten andere Studien darauf hin, dass beide Arten von Sozialkapital im Laufe der Zeit an Wirksamkeit verlieren oder erschöpft sind (Meyer, 2017). In diesem Zusammenhang sollten in künftigen Studien weitere Variablen, die für den Aufbau und die Entwicklung sozialer Beziehungen bedeutsam sind – wie z. B. Selbstvertrauen und Selbstwirksamkeit – einbezogen werden (Taylor, Davies, Wells, Gilbertson, & Tayleur, 2015).

Schließlich sollten weiterführende Analysen das Sozialkapital nicht nur auf der individuellen, sondern auch auf der Vereinsebene erfassen. Bisherige Studien aus der Desasterforschung deuten einerseits darauf hin, dass Gemeinschaften mit hohem überbrückendem Sozialkapital vergleichsweise hohe Zufriedenheitsraten z. B. mit dem Wiederaufbau der Stadt nach einem Erdbeben und schnellere Erholungsraten zu verzeichnen hatten (Nakagawa & Shaw, 2004). Eine Erosion des Brückenkapitals in Gemeinschaften kann aber auch zur Stärkung skrupelloser sozialer Netzwerke und zur Veruntreuung von Geldern für die Katastrophenhilfe führen (Masud-All-Kamal & Hassan, 2018). Ambivalent wird auch der Einfluss des Sozialkapitals auf Maßnahmen zur Eindämmung der Corona-Pandemie betrachtet. Eine starke Bindung an die Gemeinschaft und soziales Vertrauen ließen sich mit mehr COVID-19-Todesfällen in Verbindung bringen, während Familienbindung und Sicherheit mit weniger Todesfällen in Zusammenhang standen (Imbulana Arachchi & Managi, 2021). Um Mehrebenenanalysen durchzuführen, bedarf es allerdings Vereinserhebungen.

Die vorgelegten Befunde basieren auf Daten einer bevölkerungsrepräsentativen Stichprobe. Diese ermöglicht v. a. auch Einschätzungen über die Ausprägung von Sozialkapital in der Bevölkerung oder auch Vergleiche zwischen Vereinsmitgliedern und Nichtmitgliedern sowie zwischen Sportvereinsmitgliedern und Mitgliedern anderer Freiwilligenvereinigungen. Beispielsweise kann gezeigt werden, dass – von wenigen Ausnahmen abgesehen – dieselben Prädiktoren zur Vorhersage der Mitgliederbindung bedeutsam werden, wenn die Regressionsanalysen nicht nur mit Sportvereinsmitgliedern, sondern mit Mitgliedern sämtlicher Freiwilligenvereinigungen berechnet werden (Tab. 5 im Anhang).

## Anhang

**Tab. 5:** Prädiktoren der Mitgliederbindung in Freiwilligenvereinigungen als abhängige Variablen. Parameterschätzungen mit robusten Standardfehlern. OLS-Regressionen (*N* = 1385)

	Mitgliederbindung in 14 verschiedenen Vereinigungen					
	Modell 1	Modell 2a	Modell 2b	Modell 3a	Modell 3b	Modell 4
Konstante	3,093***	1,534***	1,276***	2,673***	2,687***	1,655***
Mitgliedervertrauen	–	0,147**	0,137**	–	–	0,054
Reziprozität	–	0,198***	0,218***	–	–	0,132**
Freiwilliges Engagement	–	−0,051	−0,072+	–	–	−0,073+
Gesellige Orientierung	–	−0,150**	−0,150**	–	–	−0,118*
Hilfsbereitschaft	–	0,389***	0,369***	–	–	0,277***
Ingroup-Vertrauen	–	0,134*	0,101	–	–	0,036
Outgroup-Vertrauen	–	−0,099**	−0,104**	–	–	−0,076*
Konflikte im Verein	–	–	–	−0,100**	−0,091**	−0,066*
Zufriedenheit mit Krisenmanagement	–	–	–	0,280***	0,223***	0,203***
Corona-bedingte Einschränkungen der Vereinsangebote	–	–	–	0,169**	0,203**	0,159**
Mitgliedschaftsdauer	–	–	–	0,146***	0,114*	0,108*
(Kein) Migrationsstatus	0,170**	–	0,186**	–	0,013	0,070
Geschlecht	−0,030	–	−0,019	–	0,052	0,019
Abitur	0,014	–	0,011	–	−0,034	−0,005
Kinder im Haushalt	−0,016	–	−0,026	–	−0,019	−0,033
Alter	0,006***	–	0,004**	–	0,005**	0,002
Wohnort	−0,022	–	−0,012	–	−0,126*	−0,037
(Höheres) Einkommen	0,102**	–	0,097*	–	0,011	0,054
Korr. R^2^	0,026	0,154	0,174	0,187	0,197	0,236
F	6,028	27,729	16,381	63,615	12,224	16,104

Signifikanzniveau: ****p* < 0,001, ***p* < 0,01, **p* < 0,05, +*p* < 0,10

**Tab. 6:** Pearson Korrelationen zwischen den Sozialkapitalindikatoren (ungewichtete Teilstichproben)

Alle Vereinsmitglieder → ↓ Sportvereinsmitglieder	1	2	3	4	5	6	7
1 freiwilliges Engagement^a^	–	0,254***	0,131***	0,015	0,096**	0,232***	0,083**
2 Reziprozitätsnormen	0,237***	–	0,391***	0,216***	0,215***	0,419***	0,227***
3 Mitgliedervertrauen	0,136**	0,395***	–	0,493***	0,340***	0,339***	0,161***
4 Ingroup-Vertrauen	−0,009	0,258***	0,471***	–	0,304***	0,318***	0,278***
5 Outgroup-Vertrauen	0,134**	0,275***	0,342***	0,317***	–	0,263***	0,105***
6 Gesellige Orientierungen	0,204***	0,466***	0,366***	0,393***	0,309***	–	0,274***
7 Hilfsbereitschaft	0,082*	0,250***	0,209***	0,343***	0,136**	0,400***	–

Anmerkungen: Wertebereich 1–4 außer a (0–1); Signifikanzniveau: ****p* < 0,001, ***p* < 0,01, **p* < 0,05

## Footnotes

1 Die höheren Verlustquoten dürften u. a. mit den unterschiedlichen Erhebungs‑, Stichproben- und Berechnungsmethoden zusammenhängen.

2 Als signifikant förderliche Strukturmerkmale für stabile Mitgliedschafts- und Engagementverhältnisse erwiesen sich in den Analysen ländlich geprägte Sportvereine sowie Vereine, die Geselligkeit explizit fördern, sportlichem Erfolg jedoch eine nicht allzu große Bedeutung beimessen (Schlesinger & Nagel, 2013).

3 Die Online-Erhebung „Ehrenamtlichkeit im Sportverein während der Corona-Pandemie“ (EiS-CP) wurde mit Forschungsmitteln des Bundesinstituts für Sportwissenschaft aufgrund eines Beschlusses des Deutschen Bundestages gefördert. Kooperationspartner des Projekts ist der Deutsche Olympische Sportbund (DOSB).

4 Wird zum Vergleich auf die Bestandsdaten des DOSB (2020) Bezug genommen, so fällt der Organisationsgrad in unserer Erhebung etwas geringer aus (was u. a. daran liegen kann, dass in der DOSB-Statistik Mehrfachmitgliedschaften entsprechend mehrfach gezählt werden). Allerdings sind jüngere Sportvereinsmitglieder in unserer Befragung deutlich überrepräsentiert (durch Einbeziehung soziodemographischer Merkmale werden etwaige Verzerrungen kontrolliert).

5 Die N‑Zahl für divers ist zu gering, um diese Kategorie weiter einbeziehen zu können.

6 Dir Korrelation zwischen Alter und Mitgliedschaftsdauer liegt bei r = 0,29, *p* < 0,001.

## References

[CR1] Albrecht, F. (2018). Natural hazard events and social capital: the social impact of natural disasters. *Disasters*, *42*(2), 336–360. 10.1111/disa.12246. 28857267 10.1111/disa.12246

[CR2] Aldrich, D. P., & Meyer, M. A. (2015). Social capital and community resilience. *American Behavioral Scientist*, *59*(2), 254–269.

[CR3] Baur, J., & Braun, S. (2003). *Integrationsleistungen von Sportvereinen als Freiwilligenorganisationen*. Aachen: Meyer & Meyer.

[CR4] Baur, J., Burrmann, U., & Nagel, M. (2003a). Mitgliedschaftsbeziehungen in Sportvereinen. In J. Baur & S. Braun (Hrsg.), *Integrationsleistungen von Sportvereinen als Freiwilligenorganisationen* (S. 159–190). Aachen: Meyer & Meyer.

[CR5] Baur, J., Burrmann, U., & Nagel, M. (2003b). Solidargemeinschaftliche Kleinvereine? In J. Baur & S. Braun (Hrsg.), *Integrationsleistungen von Sportvereinen als Freiwilligenorganisationen* (S. 303–330). Aachen: Meyer & Meyer.

[CR6] Beck, T. K. (2020). Alltag im Reallabor. Pandemie und Bürgerkrieg als existentielle gesellschaftliche Krisen. *Leviathan*, *48*(3), 451–469. 10.5771/0340-0425-2020-3-451.

[CR7] Behrens, C., Emrich, E., Hämmerle, M., & Pierdzioch, C. (2017). Match-Qualität und ehrenamtliches Engagement in Sportvereinen. *German Journal of Exercise and Sport Research*, *48*, 89–98.

[CR8] Bertelsmann-Stiftung (2020). *Gesellschaftlicher Zusammenhalt in Deutschland 2020. Eine Herausforderung für uns alle. Ergebnisse einer repräsentativen Bevölkerungsstudie*. Gütersloh: Bertelsmann. 10.11586/2020046.

[CR9] Braun, S. (2003). Putnam und Bourdieu und das soziale Kapital in Deutschland. Der rhetorische Kurswert einer sozialwissenschaftlichen Kategorie. *Leviathan*, *29*, 337–354.

[CR10] Braun, S. (2014). Engagementforschung im vereins- und verbandsorganisierten Sport – Themen, Ergebnisse und Herausforderungen. In A. Zimmer & R. Simsa (Hrsg.), *Forschung zu Zivilgesellschaft, NPOs und Engagement. Quo vadis?* (S. 133–148). Wiesbaden: Springer VS.

[CR11] Braun, S., Burrmann, U., & Sielschott, S. (2021). Ressourcen der Sportvereine in Zeiten der Corona-Pandemie. *Soziale Bewegungen*, *34*(4), 576–586. 10.1515/fjsb-2021-0057.

[CR12] Breuer, C. (Hrsg.). (2017). *Sportentwicklungsbericht 2015/16. Analyse zur Situation der Sportvereine in Deutschland*. Hellenthal: Strauß.

[CR13] Breuer, C., Feiler, S., & Rossi, L. (2020). Sportvereine in Deutschland: Mehr als nur Bewegung Kernergebnisse der 7. Welle des Sportentwicklungsberichts (2017/2018) sowie ausgewählte Entwicklungen der letzten 15 Jahre. http://my.page2flip.de/15646901/19774819/19782345/html5.html#/1. Zugegriffen: 22.12.2021.

[CR14] Brouwer, R., & Nhassengo, J. (2006). About bridges and bonds: community responses to the 2000 floods in Mabalane district, Mozambique. *Disasters*, *30*(2), 234–255. 16689920 10.1111/j.0361-3666.2006.00317.x

[CR15] Burrmann, U., Braun, S., & Mutz, M. (2019). Playing together or bowling alone? Social capital-related attitudes of sport club members and non-members in Germany in 2001 and 2018. *European Journal for Sport and Society*, *16*(2), 164–186. 10.1080/16138171.2019.1620412.

[CR16] Burrmann, U., Braun, S., & Mutz, M. (2020). In whom do we trust? The level and radius of social trust among sport club members. *International Review for the Sociology of Sport*, *55*(4), 416–436. 10.1177/1012690218811451.

[CR17] Coffé, H., & Geys, B. (2007). Participation in bridging and bonding associations and civic attitudes: Evidence from Flanders. *VOLUNTAS: International Journal of Voluntary and Nonprofit Organizations*, *18*(4), 385–406.

[CR18] Cohen, J. (1988). *Statistical power analysis for the behavioral sciences* (2. Aufl.). Hillsdale: Lawrence Erlbaum.

[CR19] Delhey, J., Newton, K., & Welzel, C. (2011). How general is trust in “most people”? Solving the radius of trust problem. *American Sociological Review*, *76*(5), 786–807.

[CR2020] DOSB. (2020). Bestandserhebung 2020. https://cdn.dosb.de/user_upload/www.dosb.de/uber_uns/Bestandserhebung/BE-Heft_2020.pdf. Zugegriffen: 22.12.2020.

[CR20] Elliott, J. R., Haney, T. J., & Sams-Abiodun, P. (2010). Limits to social capital: Comparing network assistance in two New Orleans neighborhoods devastated by Hurricane Katrina. *The Sociological Quarterly*, *51*(4), 624–648. 20939128 10.1111/j.1533-8525.2010.01186.x

[CR21] Elmose-Østerlund, K., & van der Roest, J.-W. (2017). Understanding social capital in sports clubs: participation, duration and social trust. *European Journal for Sport and Society*, *14*(4), 366–386.

[CR22] Hawkins, R. L., & Maurer, K. (2010). Bonding, bridging and linking: How social capital operated in New Orleans following Hurricane Katrina. *British Journal of Social Work*, *40*(6), 1777–1793.

[CR23] Hayes, A. F., & Cai, L. (2007). Using heteroskedasticity-consistent standard error estimators in OLS regression: an introduction and software implementation. *Behavior research methods*, *39*(4), 709–722. 18183883 10.3758/bf03192961

[CR24] Horch, H.-D. (1992). *Geld, Macht und Engagement in freiwilligen Vereinigungen. Grundlagen einer Wirtschaftssoziologie von Non-Profit-Organisationen*. Berlin: Duncker & Humblot.

[CR25] Imbulana Arachchi, J., & Managi, S. (2021). The role of social capital in COVID-19 deaths. *BMC Public Health*, *21*, 434–442. 33657999 10.1186/s12889-021-10475-8 PMC7928173

[CR26] Klenk, C. (2011). *Ziel-Interessen-Divergenzen in freiwilligen Sportorganisationen. Eine akteurtheoretische Analyse der Ursachen und Auswirkungen*. Hamburg: Czwalina.

[CR27] Kwon, Y., & Seo, H. (2021). Employer-sponsored sports programs amid COVID-19. The approach of social capital. *JOEM*, *63*(4), 285–290. 33252374 10.1097/JOM.0000000000002095

[CR28] Lee, J., & Fraser, T. (2019). How do natural hazards affect participation in voluntary association? The social impacts of disasters in Japanese society. *International Journal of Disaster Risk Reduction*, *34*, 108–115.

[CR29] Masud-All-Kamal, M., & Hassan, S. M. (2018). The link between social capital and disaster recovery: evidence from coastal communities in Bangladesh. *Nat Hazards*, *93*, 1547–1564.

[CR30] Meyer, M. A. (2017). Social capital in disaster research. In H. Rodríguez, W. Donner & J. E. Trainor (Hrsg.), *Handbook of disaster research, handbooks of sociology and social research* (S. 263–286). Cham: Springer.

[CR31] Mutz, M., & Nobis, T. (2012). Leisten zivilgesellschaftliche Vereinigungen einen Beitrag zur politischen Sozialisation von Jugendlichen? *Zeitschrift für Soziologie der Erziehung und Sozialisation*, *32*(4), 340–357.

[CR32] Nagel, S. (2006a). *Sportvereine im Wandel. Akteurtheoretische Analysen zur Entwicklung von Sportvereinen*. Schorndorf: Hofmann.

[CR33] Nagel, S. (2006b). Mitgliederbindung in Sportvereinen – Ein akteurtheoretisches Modell. *Sport und Gesellschaft*, *3*, 33–56.

[CR34] Nakagawa, Y., & Shaw, R. (2004). Social capital: a missing link to disaster recovery. *International Journal of Mass Emergencies and Disasters*, *22*(1), 5–34.

[CR35] Paxton, P. (2007). Association memberships and generalized trust: a multilevel model across 31 countries. *Social Forces*, *86*(1), 47–76.

[CR36] Perck, J., Van Hoecke, J., Westerbeek, H., & Breesch, D. (2016). Organisational change in local sport clubs: The case of Flemish gymnastics clubs. *Sport, Business and Management*, *6*, 158–181. 10.1108/SBM-01-2014-0002.

[CR37] Pitsch, W., & Emrich, E. (1997). „Krise des Ehrenamts“? Eine neue Analyse alter Daten. *Sportwissenschaft*, *27*, 391–408.

[CR38] Priemer, J., Krimmer, H., & Labigne, A. (2017). *Vielfalt verstehen. Zusammenhalt stärken. ZiviZ-Survey 2017*. Essen: Edition Stifterverband.

[CR39] Putnam, R. D. (1993). *Making democracy work: civic traditions in modern Italy*. Princeton: Princeton University Press.

[CR40] Putnam, R. D. (1995). Bowling alone: America’s declining social capital. *Journal of Democracy*, *6*, 65–78.

[CR41] Putnam, R. D. (2000). *Bowling alone: the collapse and revival of American community*. New York: Simon & Schuster.

[CR42] Quintelier, E. (2008). Who is politically active: The athlete, the scout member or the environmental activist? *Acta Sociologica*, *51*(4), 355–370.

[CR43] Rump, B. (2021). Sportvereine und die Pandemie. *DOSB-Presse*, (23), 29–30. https://cdn.dosb.de/user_upload/www.dosb.de/Newsletter/DOSB-Presse/2021/23_DOSB_PRESSE_web.pdf. Zugegriffen: 22.12.2021.

[CR44] Schlesinger, T., & Nagel, S. (2013). Individuelle und strukturelle Faktoren der Mitgliederbindung im Sportverein. *Sportwissenschaft*, *43*, 90–101.

[CR45] Schlesinger, T., & Nagel, S. (2015). Does context matter? Analysing structural and individual factors of member commitment in sport clubs. *European Journal for Sport and Society*, *12*(1), 53–77.

[CR46] Schlesinger, T., & Nagel, S. (2018). Individual and contextual determinants of stable volunteering in sport clubs. *International review for the sociology of sport*, *53*(1), 101–121.

[CR47] Schmitt, N. (1996). Uses and abuses of coefficient alpha. *Psychological Assessment*, *8*(4), 350–353.

[CR48] Schüttoff, U., Pawlowski, T., Downward, P., & Lechner, M. (2018). Sports participation and social capital formation during adolescence. *Social Science Quarterly*, *99*(2), 683–698.

[CR49] Seippel, Ø. (2006). Sport and social capital. *Acta Sociologica*, *49*(2), 169–183.

[CR50] Simonson, J., Kelle, N., Kausmann, C., & Tesch-Römer, C. (2021). Freiwilliges Engagement in Deutschland. Der Deutsche Freiwilligensurvey 2019. https://www.dza.de/fileadmin/dza/Dokumente/Forschung/Publikationen%20Forschung/Freiwilliges_Engagement_in_Deutschland_-_der_Deutsche_Freiwilligensurvey_2019.pdf. Zugegriffen: 22.12.2021.

[CR52] Stolle, D. (1998). Bowling together, bowling alone: the development of generalized trust in voluntary. *Political Psychology*, *19*(3), 497–525. Special Issue: Psychological Approaches to Social Capital.

[CR51] Stolle, D. (2001). Clubs and congregations. The benefits of joining an association. In K. S. Cook (Hrsg.), *Trust in Society* (S. 202–244). Thousand Oaks: SAGE.

[CR53] Strob, B. (1999). *Der vereins- und verbandsorganisierte Sport: Ein Zusammenschluss von (Wahl)Gemeinschaften?* Münster: Waxmann.

[CR54] Taylor, P., Davies, L., Wells, P., Gilbertson, J., & Tayleur, W. (2015). A review of the social impacts of culture and sport. https://assets.publishing.service.gov.uk/government/uploads/system/uploads/attachment_data/file/416279/A_review_of_the_Social_Impacts_of_Culture_and_Sport.pdf. Zugegriffen: 22.12.2021.

[CR55] Thieme, L. (Hrsg.). (2017). *Der Sportverein – Versuch einer Bilanz*. Schorndorf: Hofmann.

[CR56] Thieme, L., Liebetreu, T., & Wallrodt, S. (2017). Gewinnung und Bindung von Vorständen im Sportverein. *German Journal of Exercise and Sport Research*, *47*(2), 133–148.

[CR57] Urban, D., & Mayerl, J. (2018). *Angewandte Regressionsanalyse: Theorie, Technik und Praxis* (5. Aufl.). Wiesbaden: Springer VS.

[CR58] Van der Meer, T., & van Ingen, E. (2009). Schools of democracy? Disentangling the relationship between civic participation and political action in 17 European countries. *European Journal of Political Research*, *48*(2), 281–308.

[CR59] Van der Roest, J.-W., van Kalmthout, J., & Meijs, L. (2016). A consumerist turn in Dutch voluntary sport associations? *European Journal for Sport and Society*, *13*, 1–18. 10.1080/16138171.2016.1153882.

[CR60] Vester, M., von Oertzen, P., Geiling, H., Hermann, T., & Müller, D. (2001). *Soziale Milieus im gesellschaftlichen Strukturwandel. Zwischen Integration und Ausgrenzung* (2. Aufl.). Berlin: Suhrkamp.

[CR61] Wollebæk, D., & Strømsnes, K. (2008). Voluntary associations, trust, and civic engagement: a multilevel approach. *Nonprofit and Voluntary Sector Quarterly*, *37*(2), 249–263.

[CR62] Zimmer, A. (2007). *Vereine – Zivilgesellschaft konkret*. Wiesbaden: Springer VS.

[CR63] Zimmer, A., Basic, A., & Hallmann, T. (2011). Sport ist im Verein am schönsten? Analysen und Befunde zur Attraktivität des Sports für Ehrenamt und Mitgliedschaft. In T. Rauschenbach & A. Zimmer (Hrsg.), *Bürgerschaftliches Engagement unter Druck? Analysen und Befunde aus den Bereichen Soziales, Kultur und Sport* (S. 269–386). Opladen: Barbara Budrich.

